# Single layer centrifugation as a method for bacterial reduction in bull semen for assisted reproduction

**DOI:** 10.1007/s11259-023-10178-y

**Published:** 2023-07-22

**Authors:** Aleksandar Cojkic, Ingrid Hansson, Anders Johannisson, Eva Axner, Jane M. Morrell

**Affiliations:** 1 Department of Clinical Sciences, Swedish University of Agricultural Sciences (SLU), Uppsala, 75007 Sweden; 2 Department of Biomedical Science and Veterinary Public Health, Swedish University of Agricultural Sciences (SLU), Uppsala, 75007 Sweden; 3 Swedish University of Agricultural Sciences (SLU), Uppsala, 75007 Sweden

**Keywords:** Bull semen, Bacterial contamination, Single-layer centrifugation, MALDI-TOF MS

## Abstract

Semen samples contain bacteria originating from the animal urogenital tract, environment, and/or contamination during semen processing, negatively affecting sperm quality by producing toxins and/or competing for nutrients in extenders. The aims of this study were to evaluate two methods of Single-layer centrifuges (SLC), high and low density colloid, as a method for bacterial removal from bull semen, and to evaluate sperm quality after treatment. In total, semen samples from 20 bulls (3 ejaculates per bull) were used in this study. Bacterial reduction was evaluated by bacterial quantification (colony forming unit - CFU/mL) while bacterial identification was performed by matrix-assisted laser desorption ionization–time of flight mass spectrometry (MALDI-TOF MS) after culturing bacteria on blood agar. Sperm motility parameters were evaluated by Computer Assisted Sperm Analyses (CASA), and sperm chromatin structure assay (SCSA) by Flow cytometry. Both, High and Low density SLC reduced number of bacteria significantly (p < 0.001) compared with control. The difference in bacterial count between High and Low SLC was also significant (p < 0.001). Furthermore, High density SLC was successful in removing almost all *Bacillus* and *Proteus* spp. Most CASA parameters were significantly improved after both treatments (p < 0.001, p < 0.01, p < 0.05). The Deoxyribonucleic acid (DNA) fragmentation index evaluated by SCSA in High (p < 0.01) and Low (p < 0.05) SLC group differed significantly compared with control. Single-layer centrifugation (SLC) with either a high or a low density colloid is a suitable method for bacterial removal in bull semen.

## Introduction

Artificial insemination (AI) is a reproductive biotechnology procedure used worldwide, especially in cattle breeding. Artificial reproductive techniques such as bull semen cryopreservation for AI is used for safe animal production, primarily aimed to control the spread of disease (Mapletoft and Hasler 2005) but also to minimize bacterial contamination by implementing a high level of hygiene during semen collection. However, bull semen collection and processing is not an aseptic process although the equipment for semen collection should be sterile. The animal’s urogenital tract and every step in sperm cryopreservation process after sperm collection can be a source of bacterial contamination (Al-Kass et al. 2020; Sannat et al. 2015). To inhibit bacterial growth and the negative effect of bacteria on spermatozoa (spz) (Eini et al. 2021), antibiotics are added to semen extenders in different combinations and concentrations. This step in semen processing is regulated by national and European Union Directives, specifically for semen used in international trade (EUR-Lex 2021). Despite these implemented measures, 11 of 30 frozen bull semen doses containing unspecified antibiotics contained viable bacteria in a Brazilian study (Zampieri et al. 2013). Some antibiotics are not effective against bacteria in bull semen and there are conflicting opinions on the effect of different concentrations of these antibiotics on spermatozoa (Gloria et al. 2014). This non-therapeutic use of antibiotics in small doses increases the risk of development of antimicrobial resistance and imposes an obligation for finding alternatives.

Alternatives for antibiotics in semen extender have been discussed previously (Morrell et al. 2022; Morrell and Wallgren 2014). Plant-based substances with known antimicrobial properties on different isolated bacteria have been proposed as an alternative but until recently, their antimicrobial effect in bull sperm had not been tested. Cojkic et al. (2023) evaluated the antimicrobial effects of three plant-based substances added in different concentrations to bull semen. The results showed that the tested substances did reduce the bacterial count but some of them had deleterious effect on sperm quality as well. Curcumin at a concentration of 5% was the only substance added to bull semen that showed a reduction in bacterial count while preserving sperm quality. In another study, adding rosmarinic acid in different concentration to bull semen to test its antimicrobial effect did not affect sperm quality but did not have any antimicrobial effect either (Hagos et al. 2020), despite being antibacterial for the bacteria in boar semen (Elmi et al. 2019). These conflicting results emphasizes that criteria need to be met before additives can be considered as antimicrobial alternatives for semen extenders (Schulze et al. 2020). The minimum requirement for antibacterial ingredients in extenders used for sperm cryopreservation are that they should not be spermatotoxic. Furthermore, sperm additives should not interfere with fertility, nor evoke antibacterial resistance, and should have high stability and activity potential at low storage temperatures, be economical and easy to apply and that they should have a broad antibacterial spectrum. All of these challenges could potentially be avoided using Single Layer Centrifugation (SLC) as a physical method of separating spermatozoa from bacteria in semen samples.

From the beginning, SLC was presented as a method for improving sperm quality by selecting the “best” spermatozoa from the ejaculate (Morrell and Rodriguez-Martinez 2010). Since then, the method has been developed and adapted for different species (Goodla et al. 2014; Jiménez-Rabadán et al. 2012; Johannisson et al. 2009; Morrell and Wallgren 2011a) and different volumes of semen samples (Morrell et al. 2011). In addition to positive effects on sperm quality, SLC showed significant results in bacterial removal from stallion (Al-Kass et al. 2019; Morrell et al. 2014), boar (Morrell and Wallgren 2011b) and dog (Dorado et al. 2013) semen but, until now, it has not been tested for removal of bacteria in bull semen.

The main aim of this study was to assess two densities of colloid for SLC (high and low) evaluating their influence on bacterial reduction in bull semen. Additional aims were to evaluate sperm quality parameters, as well as differences in the bacterial count and the occurrence of bacterial species in ejaculates collected after different collection times from same bulls.

## Materials and methods

### Experimental design

Ejaculates were collected from dairy bulls and transferred to the laboratory at the Swedish University of Agricultural Sciences. Each ejaculate was split into three parts: one control and two for SLC, one each using a high or low density colloid. After centrifugation, aliquots were removed from all SLC samples and controls for bacteriology; the samples were then stored at 4° C. Further aliquots were taken for sperm quality analysis, although it was not possible to evaluate all samples because of the time taken for the bacterial analyses. Since the focus of this study was on bacterial removal by SLC, all bacterial analyses should be performed within 2 h after centrifugation to avoid growth of bacterial contaminants. For this reason, we did not have enough time to perform analyses of sperm motility for all the samples; however we randomly selected 6 samples for motility analysis, which is the number required to detect statistical significance (Arifin and Zahiruddin 2017) from a population of this size, and 20 samples for SCSA.

### Animals, semen collection and proceeding

Semen samples were collected from 20 dairy bulls (Holstein Friesian and Swedish Red) aged 1 to 4 years, housed at VikingGenetics - Skara, Artificial Insemination Station in Sweden. Semen was collected three times during one week from each bull. The first ejaculates were collected at the beginning of the week, 96 h after previous collection, second ejaculates were collected 48 h after the first, and the third ejaculates were collected 24 h after the second. The bulls were stimulated by false mounting by a teaser; final semen collection was performed by artificial vagina following standard husbandry procedures. After semen collection, the sterile collection tube containing the ejaculate was transferred to the laboratory within one minute. Semen samples were diluted 1:1 with Andromed semen extender without antibiotics (AndroMed® CSS one-step 200mL, Minitüb GmbH, Tiefenbach, Germany). Approximately 5–10 mL of diluted semen was transported overnight to the laboratory at the Swedish University of Agricultural Sciences at 6 °C in an insulated box containing a cold pack. After arrival, sperm concentration was measured with a Nucleocounter-SP 100 (Chemometec, Allerød, Denmark) as described previously (Cojkic et al. 2023) and the sample was extended to a concentration of 69 × 10^6^ cells, according to the company’s routine practice for preparing semen for freezing, using Andromed without antibiotics. Thereafter the sample was split into three: Control (A), and High (B) and Low (C) density-SLC group.

### Colloid Centrifugation

Colloid centrifugation was carried out according to the method used by Morrell et al. (2019) for boar semen but using Bovicoll (previously known as Androcoll-B; Goodla et al. 2014) adjusted with Buffer B (Goodla et al. 2014) to provide a density of 1.104 g/mL (High) or 1.0325 g/mL (Low). Briefly, the colloid was poured into 50 mL centrifuge tubes and the extended semen sample at a sperm concentration of 69 × 10^6^/mL was carefully pipetted on top. After centrifugation at 300 g for 20 min at ambient temperature (approximately 23 °C), the supernatant was removed, the sperm pellet was aspirated into a clean tube and was resuspended in fresh AndroMed without antibiotics, to give a final volume of 7 mL.

### Bacteriological analyses – MALDI-TOF MS bacterial identification and bacterial quantification

For bacterial analyses, 1 mL of sperm sample from each treatment group (A, B and C) was mixed with the same amount of peptone diluent (1 g peptone and 8.5 g NaCl per liter Milli-Q H_2_O; autoclaved at 121 °C for 15 min). Subsequently, 1 mL of this mix was transferred to cattle blood agar plates (Statens Veterinärmedicinska Anstalt - SVA, Uppsala, Sweden) which were then incubated at 37 ± 1 °C and examined for bacterial growth after 24 h. Bacterial colonies of different macromorphologies were recultured on new blood agar plates and incubated for 24 h at 37 ± 1 °C with 5% CO_2_ to obtain a pure culture. The colonies from the pure culture were then identified at the species level by matrix-assisted laser desorption ionization–time of flight mass spectrometry (MALDI-TOF MS) (Bruker Daltonics, Billerica, MA, USA). The mass spectrum of bacterial isolates was compared with those of known bacterial strains in the database (Bruker Daltonics, Billerica, MA, USA). Score values between 2.0 and 3.0 were considered accurate at both genus and species levels, whereas score values between 1.7 and 2.0 were considered reliable only at the genus level. If colonies could not been identified at the first attempt, a second attempt was made. If classification was still not possible they were recorded as belonging to the “no identification possible” group.

The total number of viable aerobic bacteria in the semen samples was analyzed according to the standard protocol (NMKL 86, 5 Ed., 2013), with slight modifications. In brief, 1 mL of each sperm sample was transferred to 1 mL of diluent (the same diluent used for culturing bacteria on blood agar). The samples were homogenized using a vortex (Saveen and Werner, Malmö, Sweden). A 1.0 mL of diluted sample was pour plated into a petri dish measuring 9 cm in diameter and mixed with 10–15 mL plate count agar (PCA) (Oxoid, Basingstoke, UK) melted in boiled water and thereafter placed in a 48 °C water bath to keep it liquid until required. The petri dishes were moved gently to distribute the bacteria evenly in the agar plates. When the agar in the petri dishes had solidified, a further 10–15 mL of PCA was added to each dish as an overlay to avoid swarming and facilitate enumeration. After solidification of the agar, the plates were incubated at 30 °C for 72 h. Each sample was evaluated in duplicate. The total number of colony forming unit (CFU) was calculated using a colony counter (Gerber instruments, Im Langhag, Switzerland). The standard ISO 7218:2007/A1:2013 formula was used for calculating CFU/mL which was Log transformed and presented as Log10 CFU/mL. Bacteriology analyses were performed on all ejaculates, in total 60 samples, three from each of the 20 bulls.

### Sperm quality analyses – computer assisted sperm analyses (CASA) and sperm chromatin structure assay (SCSA)

The sperm concentration varied between the samples; the minimum and maximum number of spermatozoa per sample for the High group were 18.38 and 217.70 million spz, respectively, and for the Low group were 28.99 and 604.24 million spz, respectively. Motility evaluation was performed using a SpermVision analyzer (Minitüb GmbH, Tiefenbach, Germany) connected to a Zeiss microscope with a heated stage (38 °C). Semen samples were equilibrated to room temperature for at least 10 min, before motility analysis. Sperm motility was analyzed in eight fields (at least 850 spermatozoa in total) using the SpermVision software program version 3.8 with settings adjusted for bull spermatozoa, in a 5-µL aliquot of the semen sample on a warm slide covered with an 18 × 18 mm cover slip (VMR, Leuven, Belgium). The following parameters were used to analyze motility: total motility (TM, %), progressive motility (PM, %), curvilinear velocity (VCL, µm/s), average path velocity (VAP, µm/s), straight line velocity (VSL, µm/s), linearity (LIN, as VSL/VCL), straightness (STR, as VSL/VAP), wobble (WOB, as VAP/VCL), beat cross frequency (BCF, Hz), and amplitude of lateral head displacement (ALH, µm). Images were obtained at 200 × magnification using a phase contrast microscope. Particles with an area ranging from 20 to 100 µm^2^ were identified as cells and were included in the analysis. Spermatozoa were considered as immotile if the area under curve (AOC) was < 5, BCF < 0.2, and VSL < 0.2; they were considered to be locally motile if spermatozoa covering a straight-line distance (DSL) was < 4.5. In total, semen samples from six bulls, one ejaculate per bull were evaluated for motility.

Chromatin integrity was analysed using the sperm chromatin structure assay (SCSA). Flow cytometric (FC) analysis was performed using a FACSVerseTM flow cytometer (BD Biosciences, Becton Dickinson and Company, San Jose, CA, USA). The assay uses the metachromatic dye acridine orange (AO) to assess the susceptibility of sperm deoxyribonucleic acid (DNA) to acid-induced denaturation. The DNA fragmentation index (%DFI) was calculated and expressed as the proportion of cells with a high ratio of denatured, single stranded DNA; %DFI = (red fluorescence/[green fluorescence + red fluorescence]) × 100. The procedure, media preparation, buffers, and solutions used in the assay have been described in detail previously (Januskauskas et al. 2003). Briefly, 50 µL of the semen sample was mixed with the same amount of Tris-sodium chloride and ethylene-diaminetetraacetic acid buffer (TNE buffer) and immediately transferred to a liquid nitrogen container for snap-freezing. The samples were stored at − 80 °C until analysis. They were thawed on ice, an aliquot (10 µL) was mixed with 90 µL of TNE, and 200 µL of acid-detergent solution. After 30 s, the sample was stained with 600 µL of AO staining solution. The stained samples were analyzed within 3–5 min of AO staining. The data obtained in the sperm chromatin structure assay were further analyzed using FCS Express 5 software (De Novo, Glendale, CA, USA). In total, semen samples from 20 bulls (one ejaculate per bull) were used for SCSA analyses.

### Statistical analyses

The statistical program IBM Statistic SPSS 26 was used to analyse the data of this study. Differences in CASA parameters, SCSA results between groups (Control, High and Low density SLC) and bacterial count between ejaculates were analysed by Kruskal Wallis test followed by Dunn`s post-hoc test since the sample size was low for a parametric test to be used. Furthermore, repeated measures two-way ANOVA followed by Tukey´s multiple comparisons test was used to calculate the differences in bacterial number (Log10 CFU/mL) between SCL groups and control. Three levels of significance have been used (p < 0.05, p < 0.01 and p < 0.001); the data are presented as mean ± standard deviation (SD). Descriptive statistics were used to present bacteriological results, for bacteria identified by MALDI-TOF MS between bulls and ejaculates.

## Results

In total, 49 bacterial species were identified (Table 1). The results are presented for individual ejaculates and groups (A, B and C) to show the variation in appearance of different bacterial species. Only eight bacterial species appeared in all ejaculates, while 11, 10 and 9 species were identified on only one occasion, i.e. first, second or third ejaculate, respectively. The total bacterial count differed between ejaculates. The highest number of different macromorphological bacterial colonies was present in first ejaculates (n = 90) collected after 96 h of abstinence, followed by second (n = 75) and third (n = 73) ejaculates where the period of abstinence was shorter (48 and 24 h, respectively). Furthermore, the differences in total number of species differed between groups. The number of bacteria classified in “no identification possible” increased with decreased time of abstinence (92 h for the first ejaculate, 48 h for the second, and 24 h for the third ejaculate), and with the number of species that could be identified.

**Table 1:** Bacterial species identified in bull semen samples by MALDI-TOF MS between ejaculates and different colloids sample groups

	**First ejaculate**			Second ejaculate			Third ejaculate		
	A	B	**C**	A	B	**C**	A	B	C
*Alkalihalobacillus clausii*	1	0	1	1	0	0	2	4	1
*Bacillus licheniformis*	7	1	3	1	0	2	6	0	3
*Bacillus pumilus*	5	1	6	9	0	0	3	0	1
*Corynebacterium xerosis*	7	0	1	1	0	0	5	0	1
*Enterobacter cloacae*	1	0	0	2	0	0	2	0	2
*Micrococcus luteus*	6	0	0	0	3	6	1	2	2
*Proteus mirabilis*	4	0	0	11	0	5	3	0	0
*Staphylococcus sciuri*	1	0	0	4	0	0	5	0	3
*Bacillus subtilis*	0	0	1	0	0	1			
*Histophilus somni*	7	0	0	6	0	2			
*Staphylococcus aureus*	3	0	0	1	0	0			
*Bacillus cereus*	1	1	2				4	1	0
*Corynebacterium freneyi*	1	0	1				1	0	1
*Proteus vulgaris*	1	0	0				1	0	0
*Staphylococcus warneri*	1	0	0				0	1	0
*Actinobacillus seminis*	3	0	1						
*Aerococcus viridans*	0	0	1						
*Agrobacterium radiobacter*	8	0	4						
*Agromyces mediolanus*	0	2	3						
*Gordonia rubripertincta*	1	0	1						
*Microbacterium maritypicum*	0	0	1						
*Rothia kristinae*	1	0	0						
*Sphingomonas paucimobilis*	0	1	0						
*Solibacillus silvestris*	1	0	0						
*Staphylococcus simulans*	2	0	0						
*Streptococcus uberis*	1	0	0						
*Ureibacillus thermosphaericus*	1	0	0						
*Enterobacter bugandensis*				1	0	0	0	1	0
*Staphylococcus chromogenes*				1	1	0	2	0	0
*Staphylococcus epidermidis*				0	1	0	0	0	1
*Bacillus marisflavi*				0	1	0			
*Bacillus megaterium*				1	0	0			
*Bacillus siralis*				0	0	0			
*Corynebacterium sanguinis*				1	0	0			
*Microbacterium ginsengiterrae*				2	0	0			
*Micrococcus lylae*				0	0	0			
*Paenibacillus amylolyticus*				1	0	0			
*Paenibacillus lactis*				0	1	0			
*Streptococcus parasanguinis*				0	1	0			
*Trueperella pyogenes*				2	0	1			
*Corynebacterium cystitidis*							3	0	1
*Escherichia coli*							1	0	1
*Neisseria subflava*							1	1	0
*Nosocomiicoccus massiliensis*							1	0	0
*Proteus hauseri*							1	0	0
*Staphylococcus capitis*							0	1	0
*Staphylococcus hominis*							0	2	1
*Staphylococcus pasteuri*							1	0	0
*Streptococcus mitis*							0	1	0
No identification possible	28	8	13	31	6	11	34	6	17
Total Identified Species	62	6	28	44	8	18	39	14	22
Total Bacterial Number	90	14	41	75	14	29	73	20	39

**Abbreviations**: A – Control group of semen samples, B – High density colloid treatment; C – Low density colloid treatment.

**Fig. 1:**
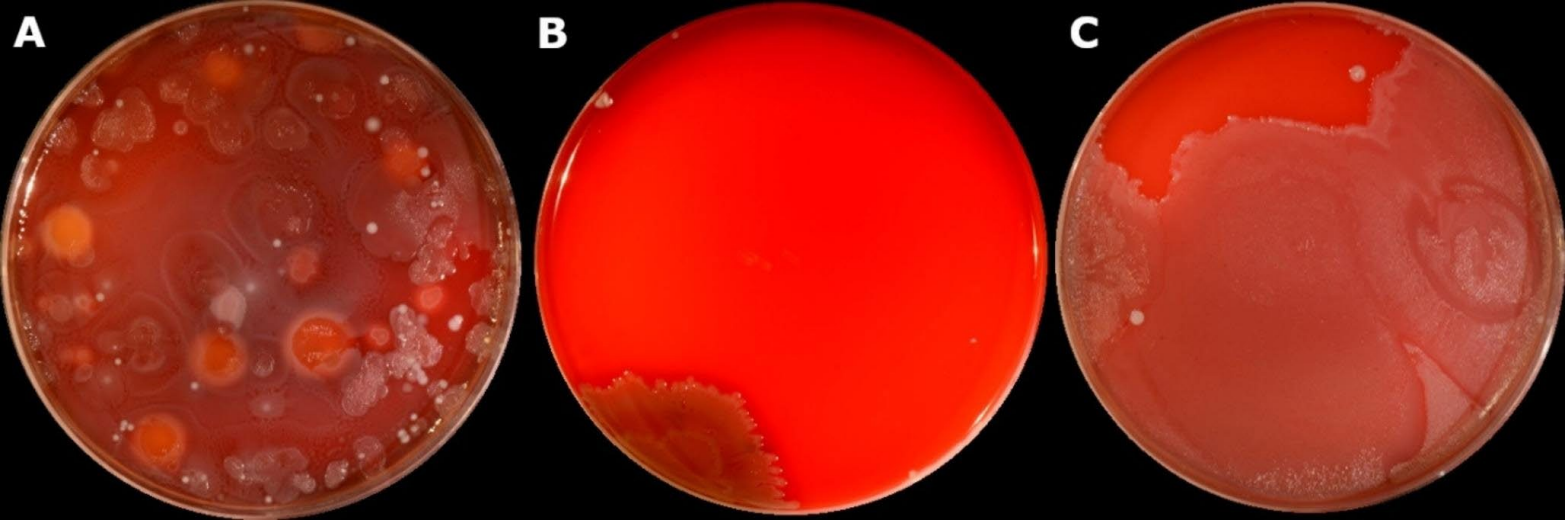
Growth of bacteria from bull semen samples on blood agar plates after 1 day of incubation in 37 °C. Plate **A** represents Control semen sample, while plates **B** and **C** represent semen sample after High and Low Colloid centrifugation, respectively

The level of reduction in different bacterial species differed between Control (A) and High (B) and Low (C) density group, where High density colloid was successful in removing almost all *Bacillus* spp. and *Proteus* spp. (Fig. 1).

On the individual bull level (Table 2), there were differences in the number of macromorphological different bacterial colonies between bulls and ejaculates of the same bull. The high density colloid was more successful in total bacterial removal from the second and third ejaculates in the majority of bulls (10/20 and 8/20, respectively) compared to the first one (5 of 20 bulls).

**Table 2:** Number of macromorphological different bacterial colonies between bulls and ejaculates

	First ejaculate			Second ejaculate			Third ejaculate		
	A	B	C	A	B	C	A	B	C
Bull 1	3	3	1	7	NG	5	4	NG	2
Bull 2	2	1	NG	2	NG	NG	3	2	NG
Bull 3	3	1	2	5	NG	NG	7	NG	7
Bull 4	4	3	3	2	NG	NG	3	NG	1
Bull 5	6	1	1	3	2	NG	NG	NG	NG
Bull 6	2	1	2	5	NG	2	3	1	2
Bull 7	5	NG	7	7	2	4	1	1	NG
Bull 8	4	5	4	9	1	2	1	1	NG
Bull 9	7	1	7	2	2	3	11	1	3
Bull 10	2	1	NG	2	NG	1	6	2	5
Bull 11	4	1	2	3	NG	3	3	NG	NG
Bull 12	5	2	2	6	NG	NG	2	NG	NG
Bull 13	5	NG	NG	7	2	2	2	1	0
Bull 14	3	NG	2	2	1	1	1	1	0
Bull 15	8	2	8	4	2	5	7	4	10
Bull 16	3	2	5	NG	1	NG	5	4	NG
Bull 17	2	NG	2	3	NG	NG	4	2	3
Bull 18	3	NG	NG	1	NG	NG	3	1	1
Bull 19	5	1	2	1	1	NG	6	NG	5
Bull 20	4	2	3	3	4	2	7	NG	NG

**Abbreviations**: A – Control group of semen samples, B – High density colloid treatment, C – Low density colloid treatment, NG – no bacterial growth.

**Fig. 2:**
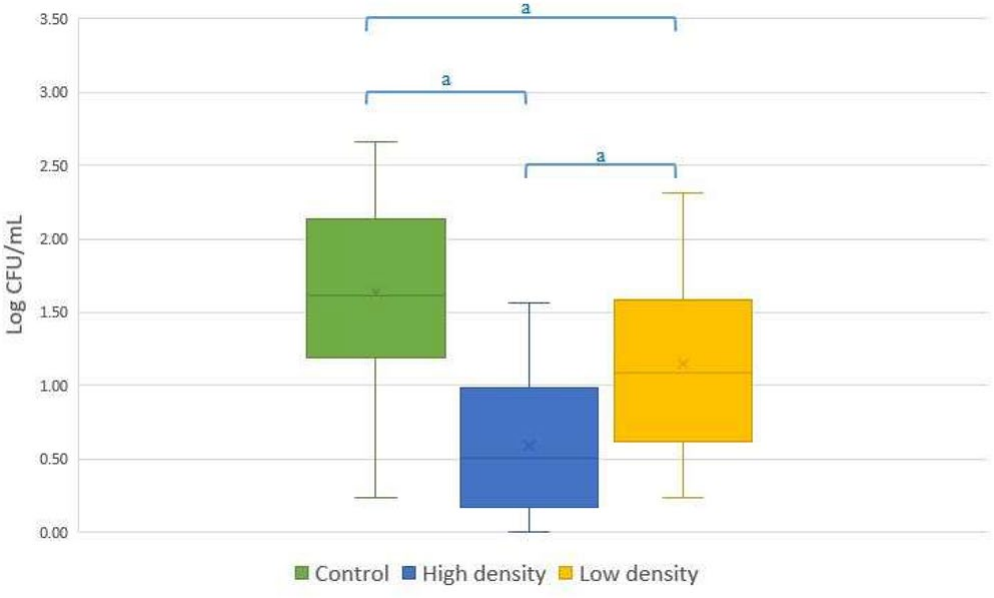
The number of bacteria (Log10 CFU/mL) in semen samples from Control, High density and Low density group. The letter a, denote significant differences between colloids at p < 0.001

Bacterial count differed significantly between the groups (Fig. 2). The lowest number of bacteria was observed in the High density group where some of the samples did not show any bacterial growth. However, it should be noted that the number of viable aerobic bacteria (CFU/mL) was low from the beginning; 90% of the bulls had an average bacterial count lower than 200 CFU/mL and 60% lower than 100 CFU/mL before samples were processed by SLC.

**Fig. 3:**
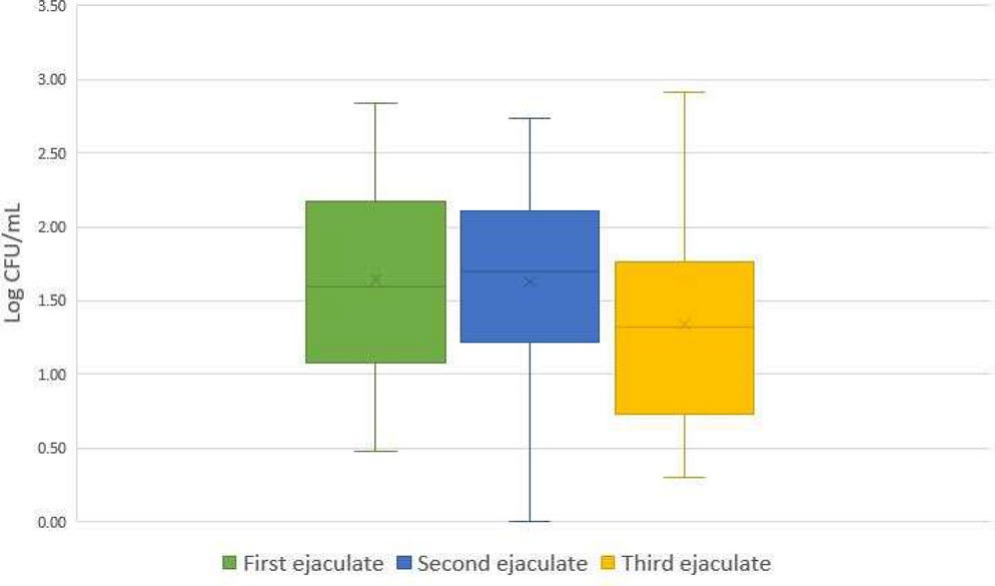
Bacterial count (Log10 CFU/mL) between first, second and third ejaculates (92 h, 48 h and 24 h, respectively)

Furthermore, the number of bacteria in the first, second and third ejaculates showed a trend towards significance between the first and third ejaculates (p = 0.07), and the second and third ejaculates (p = 0.06). Thus, the lowest number was present in third ejaculate, with the shortest time interval (24 h) between two semen collections (Fig. 3).

There were significant differences in kinematics in almost all CASA parameters between groups (Table 3). For STR and WOB, significant difference was observed between Control and High density colloid SLC group. Only LIN did not show differences between any treatments.

**Table 3:** CASA parameters of bull semen samples after colloid centrifugation (Mean ± SD)

	Control (*n* = 6)	High density (*n* = 6)	Low density (*n* = 6)
**Total motility**	40.39 ± 14.34^a^	80.04 ± 10.33^ac^	51.86 ± 15.18^c^
**Progressive motility**	31.99 ± 13.64^a^	69.75 ± 10.36^ac^	42.66 ± 16.96^c^
**VAP**	33.33 ± 3.49^ac^	71.21 ± 9.88^a^	61.38 ± 9.20^c^
**VCL**	56.35 ± 5.61^ac^	137.99 ± 18.77^a^	116.57 ± 25.32^c^
**VSL**	20.79 ± 3.65^ac^	60.16 ± 10.70^a^	47.91 ± 7.91^c^
**STR**	0.62 ± 0.10^b^	0.83 ± 0.04^b^	0.78 ± 0.06
**LIN**	0.37 ± 0.07	0.43 ± 0.06	0.42 ± 0.09
**WOB**	0.59 ± 0.05^c^	0.51 ± 0.05^c^	0.53 ± 0.06
**ALH**	2.92 ± 0.33^bc^	4.07 ± 0.41^c^	4.36 ± 1.24^b^
**BCF**	17.28 ± 0.95^ac^	29.08 ± 2.54^c^	25.48 ± 2.68^a^

**Abbreviations**: TM—Total motility, PM—Progressive motility, VCL—curvilinear velocity, VAP—average path velocity, VSL—straight line velocity, LIN—linearity, STR—straightness, WOB—wobble, BCF—beat cross frequency, ALH—amplitude of lateral head displacement, SD – Standard deviation, *n* – number of samples; Superscript letters within a row denote significant differences between groups of samples: a—p < 0.001; b—p < 0.01; c—p < 0.05

**Fig. 4:**
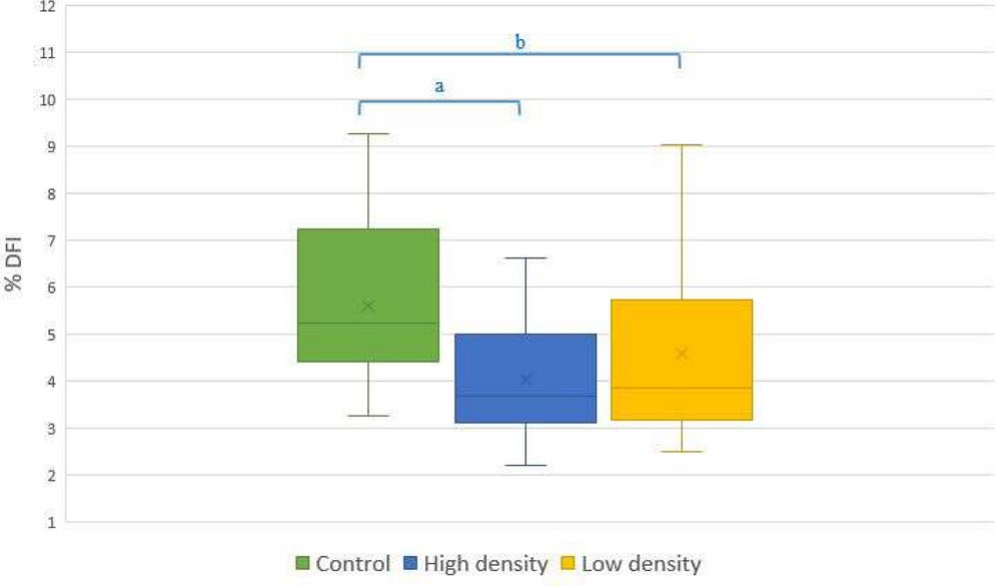
The SCSA results (%DFI) Control, High and Low density colloid groups. In total, semen samples form 20 bulls were used, one ejaculate per bull. The letters denote significant differences between groups of sample a – p < 0.01; b – p < 0.05

A decrease in the proportion of spermatozoa with damaged chromatin integrity was observed after SLC (Fig. 4).

There was a significantly lower proportion of spermatozoa with damaged chromatin in both SLC groups compared with control, while no significant differences was observed between High and Low sperm treatments.

## Discussion

The aim of this study was to evaluate two densities of colloid, High and Low, for SLC as a physical method for bacterial removal from bull semen samples. Additional aims were to evaluate selected sperm quality parameters after SLC and to identify differences in number and appearance of bacterial species in ejaculates collected at different times, from the same group of bulls. Colloid centrifugation resulted in a significant reduction in the number of bacteria in the sperm samples. Samples after either High or Low SLC had a lower number of bacteria than the controls. Furthermore, the number of bacteria differed between ejaculates collected after different periods of abstinence, between individual bulls and between SLC treatments.

The number of bacteria and bacterial species identified differed between different ejaculates within bull and between individual bulls. Since government directives stipulate that antibiotics should be added to the semen extender used for semen cryopreservation, specifically for international trade, few studies have been conducted to evaluate the seminal microbiota of healthy bulls. Results from the metagenomic analyses of bull semen microbiome in healthy animals were correlated with sperm quality (Medo et al. 2021) and fertility (Cojkic et al. 2021). However, the viability of bacteria identified by this method is unknown, and identification of bacteria to genus level has some degree of uncertainty. Identification of viable bacteria by MALDI-TOF MS represent an effective, objective, and time-saving method for identification of viable bacterial species compared with traditional biochemical characterization after culture. Eight bacterial species were identified in all the ejaculates. Similarly, González-Marín et al. (2011) showed that some bacterial strains can be found repeatedly in separate ejaculates from the same bull. The bacterial species which were present in all ejaculates were the same species that were identified in a study by Mitra et al. (2016) on commercial frozen bull semen. Apparently, these bacteria survived the cryopreservation process even with antibiotics in the semen extender. The bacteria genera that were isolated in all ejaculates in the present study were *Bacillus* spp., *Corynebacterium* spp., *Micrococcus* spp., *Proteus* spp., and *Staphylococcus* spp. Among the isolated bacteria, *Bacillus licheniformis* was previously identified as a cause of late abortion in cattle with evidence of placental lesions (Cabell 2007). Furthermore, *Micrococcus luteus* is part of the natural human skin flora (Young et al. 2010) but was also isolated from frozen bull semen samples (Abro et al. 2009). *Corynebacterium xerosis* was associated with bovine mastitis (Gonçalves et al. 2014); however, it is recommended that MALDI-TOF MS should be used as an alternative method for species-level diagnoses of bovine intramammary infections caused by *Corynebacterium* spp. Five isolates of *Corynebacterium* spp. (2.8%) were not correctly identified at species level with MALDI-TOF MS and two isolates (1.1%) were considered as unidentified. *Proteus* spp. together with several other bacterial species, have been defined as important opportunistic pathogens associated with several reproductive problems in cattle, presenting with severe vaginal purulent discharge and odor (McDougall et al. 2011). However, in other studies *Proteus mirabilis*, together with *Proteus vulgaris* were the most commonly isolated bacteria from environments contaminated with calf feces and urine from healthy cattle (Hawkey et al. 1986). *P. mirabilis* isolates were characterised as emerging multiresistant bacteria decades ago (Nikolnikov 1988) based on the presence of two of four plasmids responsible for carrying the resistance gene in 85% of the isolates. *P. mirabilis* has been identified as a common bacteria causing complicated urinary tract infection in humans, sometimes with bacteremia (Chen et al. 2012). However, the results of our study show that SLC with a High density colloid was successful in removing bacteria, with almost complete elimination of *Bacillus* and *Proteus* spp. (Tables 1; Fig. 1).

An additional aim of this study was to evaluate the influence of semen collection interval (time between two semen collections) on bacterial number and diversity. The bacterial count differed between the bulls and ejaculates although the number of CFU/mL was low from the beginning. This is in accordance with a previous study on Swedish dairy bulls from the same bull station (Cojkic et al. 2023) which confirms that semen collection in this bull station has continuously strict hygiene measures. Although the number of CFU/mL was low from the beginning, the bacterial reduction after centrifugation was significant for both colloids compared with the controls and also differed significantly between the High and Low density colloid SLC groups. This is in accordance with previous studies in stallions (Morrell et al. 2014), boars (Morrell and Wallgren 2011b) and dog (Luño et al. 2020) where SLC was used for evaluation of bacterial reduction. However, in the stallion study, the semen samples were experimentally contaminated with known numbers of selected bacteria; more than 90% of bacteria were removed with this method (Morrell et al. 2014). In the study with boar semen (Morrell and Wallgren 2011b) six out of ten samples showed complete removal of bacterial contaminants after SLC treatment, which is slightly lower than the results of this study. However, SLC with a High density colloid in this study showed better results compared with Low density SLC in bacterial reduction.

The proportion of the original bacterial load remaining depended on how soon after collection the centrifugation was performed but also on the type of bacteria present and the original bacterial load (Morrell and Wallgren 2011a). Further studies with boar semen showed that a substantial reduction in bacterial count could be achieved with low density colloids at either 40% (Morrell et al. 2019), or 30 and 20% (Deori et al. 2020). In the latter study, more spermatozoa could be retrieved with the 20% colloid than with the 30% colloid. In preliminary studies with bull semen, 20% Bovicoll was tested but the extended semen mixed with the colloid immediately, despite careful layering. The lowest density of colloid that could be used with bull semen without mixing was 25%, hence the choice of the colloid with density 1.0325 g/mL as the Low SLC in the present study.

Sperm quality (motility and %DFI) was also improved by SLC, with a greater improvement being observed in the high treatment group. Both High and Low treatment groups had significantly higher values of CASA kinematics except LIN, compared with control samples and between SLC groups. The highest CASA values were found in the High SLC group, followed by the Low group, and lastly control. These results differ from a previous experiment with bull semen (Goodla et al. 2014) where total and progressive motility were not different between SLC and Control. However, the sample size in the present study (n = 6) was lower than in the study by Goodla et al. (2014) (n = 60). The SCSA results in our study showed that both SLC treatments reduced the proportion of sperm cells with damaged chromatin compared with control. However, there was no difference between High and Low treatment groups. The presence of bacteria in commercial bull semen samples was associated with increased DNA fragmentation (González-Marín et al. 2011). Their study showed that chromatin damage was correlated with bacterial growth and differed between bulls.

Natural mating is not an aseptic process and the female reproductive tract has a well-developed response to cope with bacteria introduced during mating. In most cases, this response is able to cope with the bacterial load in semen but if certain bacteria are present or the bacterial numbers are too high, infection may result. Based on our results, SLC could be an alternative method for removal of bacteria from semen samples and selection of spermatozoa with intact chromatin structure, since the bacterial removal was almost complete and DNA fragmentation was low. A similar result was observed in a recent study on SLC using a low density colloid with boar semen (Lacalle et al. 2023).

In conclusion, Single-layer centrifugation with either a high or a low density colloid is a suitable method for bacterial removal, where SLC with a High density colloid showed almost complete bacterial removal and selection of spermatozoa of best quality. Furthermore, High density SLC completely removed potentially pathogenic bacteria species such as *Bacillus* spp. and *Proteus* spp., presenting as a good alternative for antibiotics as a tool against development of antimicrobial resistance.

## Data Availability

Not applicable.
